# Self-Assembling Protein Surfaces for *In Situ* Capture of Cell-Free-Synthesized Proteins

**DOI:** 10.3389/fbioe.2022.915035

**Published:** 2022-07-07

**Authors:** Ella Lucille Thornton, Sarah Maria Paterson, Zoe Gidden, Mathew H. Horrocks, Nadanai Laohakunakorn, Lynne Regan

**Affiliations:** ^1^ Centre for Synthetic and Systems Biology, Institute of Quantitative Biology, Biochemistry and Biotechnology, School of Biological Sciences, University of Edinburgh, Edinburgh, United Kingdom; ^2^ School of Chemistry, University of Edinburgh, Edinburgh, United Kingdom

**Keywords:** cell-free (CF) protein synthesis, surface immobilization, self-assembling, covalent attachment, protein-based tools

## Abstract

We present a new method for the surface capture of proteins in cell-free protein synthesis (CFPS). We demonstrate the spontaneous self-assembly of the protein BslA into functionalizable surfaces on the surface of a CFPS reaction chamber. We show that proteins can be covalently captured by such surfaces, using “Catcher/Tag” technology. Importantly, proteins of interest can be captured either when synthesised *in situ* by CFPS above the BslA surfaces, or when added as pure protein. The simplicity and cost efficiency of this method suggest that it will find many applications in cell-free-based methods.

## Introduction

The ability to immobilise proteins on surfaces facilitates the separation of the protein from all solution constituents, and therefore has a multitude of useful applications, including for protein purification, biosensing, and continuous flow enzymatic catalysis (Berrade et al., 2011).

Functional display of macromolecules on a surface, however, is difficult to achieve. Non-specific adherence of a molecule to the surface may cause it to denature, and non-specific orientation of presentation may make the active site of a molecule inaccessible (Kim and Herr, 2013; de Marco, 2018; Yang et al., 2018).

Cell-free protein synthesis (CFPS) is a powerful method to generate proteins *in vitro* using biochemical reactions. The process involves combining enzymatic machinery required for transcription and translation with an appropriate mix of small molecules and a DNA template encoding the protein of interest. The technique is now widely applied within synthetic biology for protein production, but also for diverse applications including biosensing, diagnostics, and materials production (Kelwick et al., 2020; Laohakunakorn et al., 2020). Much work has gone into improving the yield and lifetime of CFPS systems generated from a variety of organisms, with the best performing examples capable of producing more than 4 mg/ml of protein over 20 h in batch-mode reactions (Garenne et al., 2021).

Despite many advances in the optimisation of the CFPS reaction itself, there have been far fewer developments in surface capture technologies (Cole et al., 2020; Banks et al., 2022).

Surface immobilisation of proteins expressed in cell-free reactions was demonstrated in 2001, with a method named PISA (Protein *in situ* Array) (He and Taussig, 2001). In PISA, immobilisation of a His-tagged protein of interest, produced by CFPS, is achieved by attachment to a Ni-NTA coated surface. This method has the advantage of site-specific spontaneous immobilisation. It is limited by the relatively low strength of the Ni-NTA – His-tag interaction.

Subsequently, cell-free expression from immobilised DNA and capture of the protein product on the surface was reported in the method named NAPPA (Nucleic Acid Programmable Protein Array) (Ramachandran et al., 2004). In NAPPA, biotinylated DNA is immobilised on a surface coated with a mixture of avidin and polyclonal anti-GST antibodies. The latter are used to capture GST-tagged proteins, produced by CFPS. This method is powerful because both the DNA template and the protein product are attached to the surface. A limitation of this method is the heterogeneous orientation of the GST (Glutathione S-Transferase) antibodies on the surface, and the range of affinities of the polyclonal antibodies for the GST-tagged protein.

More recently, advanced protocols have been developed and implemented (Manzano-Román and Fuentes, 2019) including the MITOMI (mechanically-induced trapping of molecular interactions) method, which implements the NAPPA protocol in a microfluidic setting, at high throughput (Maerkl and Quake, 2007). In MITOMI, anti-His antibodies, biotinylated at multiple positions, are attached to a multi-layer surface of biotinylated BSA coated with neutravidin. This enables the pull-down of His-tagged protein products generated in CFPS. MITOMI has been used to characterise the binding energy landscape of transcription factors, as well as kinetic rate constants (Geertz et al., 2012).

The desirable features of a surface capture method for use with CFPS include: ease of assembly, homogeneity of surface presentation, retention of activity of the immobilised molecule, robust attachment, and cost effectiveness (Kilb et al., 2014). Here we describe a new system that we believe meets all desirable criteria.

We form a protein-based surface using the protein BslA, a small (15 kDa) amphiphilic protein that self-assembles at hydrophobic/hydrophilic interfaces (Hobley et al., 2013). BslA is capable of surface formation on the surface of hydrophobic glass, as shown previously by TEM (Bromley et al., 2015). We covalently link proteins to the BslA surface using Tag/Catcher technology, where a short peptide, SpyTag or SnoopTag, spontaneously forms a covalent bond with its cognate protein partner, SpyCatcher or SnoopCatcher, when mixed (Veggiani et al., 2016).

We have previously shown that fusing the hydrophilic end of BslA to a short peptide tag does not interfere with its ability to form surfaces. Thus, by fusing BslA to either SpyTag or SnoopTag peptides, we can capture a protein of interest (POI) that is fused to the cognate SpyCatcher or SnoopCatcher protein (Schloss et al., 2016).

SpyTag/SpyCatcher and SnoopTag/SnoopCatcher technology is especially well-suited for surface attachment applications because it is specific, rapid, and genetically encodable (Zakeri et al., 2012; Veggiani et al., 2016; Lange and Polizzi, 2021). The covalent linkage of the Tag/Catcher pairs is robust, and the availability of two orthogonal peptide/protein pairs enables different proteins to be attached to the surface. We show that specific surface capture occurs from a complex mixture of proteins. We also show that these surfaces can capture either proteins synthesised in the cell-free reaction, or purified proteins added to the cell-free reaction.

## Results

### Spontaneously Self-Assembled BslA Protein Surfaces can be Functionalised by Covalent Attachment of Proteins

We have previously demonstrated BslA-Tag assembly and functionalisation, using Langmuir-Schaeffer deposition followed by incubation with a purified fluorescent protein (FP) fused to the appropriate Catcher protein (Williams et al., 2018). Here we show that a simpler, one-step, self-assembly method can also be used, and demonstrate that it is compatible with CFPS.

Specifically, we show that when an aqueous solution of wild-type (WT) BslA, or the fusion proteins BslA-SpT (BslA-SpyTag) or BslA-SnT (BslA-SnoopTag) are incubated with a hydrophobic glass slide, they spontaneously self-assemble into surfaces, which can be functionalised using the Tag/Catcher technology.

**FIGURE 1:**
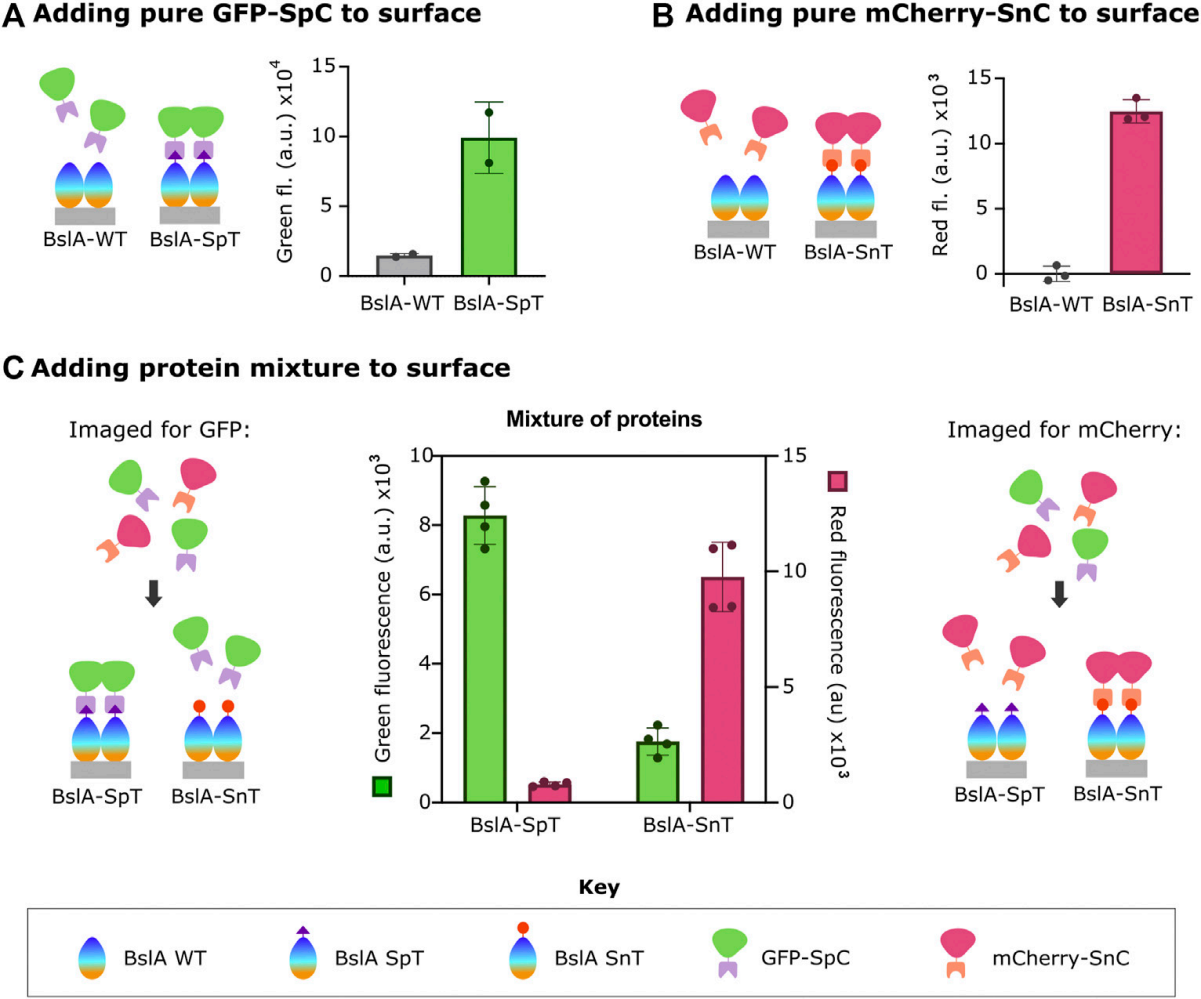
Surfaces formed by BslA-SpT or BslA-SnT capture FP fused to SpC or SnC, respectively. The key at the bottom of the figure indicates the identity of the protein components in the cartoons. In A-C, the cartoons left illustrate the experiment in schematic form, with the corresponding data shown next to it. Purified **(A)** GFP-SpC or **(B)** mCherry-SnC proteins were incubated with BslA-WT, BslA-SpT or BslA-SnT surfaces. Fluorescence remaining in each well was measured after removal of non-bound protein and washing of the wells. In **(C)**, a similar protocol was followed, but here mixtures of GFP-SpC and mCherry-SnC were incubated (at equimolar concentration) with either BslA-SpT or BslA-SnT surfaces. Measured green fluorescence of either the BslA-SpT or BslA-SnT coated wells are shown with the green coloured bars, corresponding to the left *y*-axis. Measured red fluorescence of the wells is shown with the pink coloured bars, with values corresponding to the right *y*-axis. In all cases, the fluorescence of a well with BslA and buffer was subtracted as the blank. Original data is available in the Supplementary Data Sheet. Error bars show standard deviation.

Figure 1 shows these data. After surface formation, the surface was incubated with fluorescent protein-Catcher fusion proteins (FP-Catcher), where FP is either mCherry or GFP. Unbound FP-Catcher was washed away, and the fluorescence retained on the surface was measured. We observe a significant fluorescent signal for both GFP-SpC incubated with BslA-SpT surfaces, and mCherry-SnC incubated with BslA-SnT surfaces indicating specific attachment to the surface via Tag/Catcher interaction.

By contrast, when either FP-Catcher protein is incubated with a BslA-WT surface, fluorescence is not retained, indicating that there is little non-specific binding of the fusion proteins to BslA-WT.

We also observed that when a mixture of mCherry-SnC and GFP-SC is incubated with a BslA-SnT surface, only mCherry-SnC is captured. Conversely, when a mixture of mCherry-SnC and GFP-SC is incubated with a BslA-SpT surface, only GFP-SC is captured (Figure 1C). In summary, the amount of either protein that is specifically captured is not diminished by the presence of the non-cognate protein. Additionally, we found that proteins specifically bound to the BslA surface were not affected by washing with additives such as BSA (Bovine Serum Albumin) or TWEEN20 (Supplementary Figure S3).

Following the observations described in Figure 1, we sought to determine if mCherry-SpC is covalently attached to the BslA-SnT surface.

**FIGURE 2:**
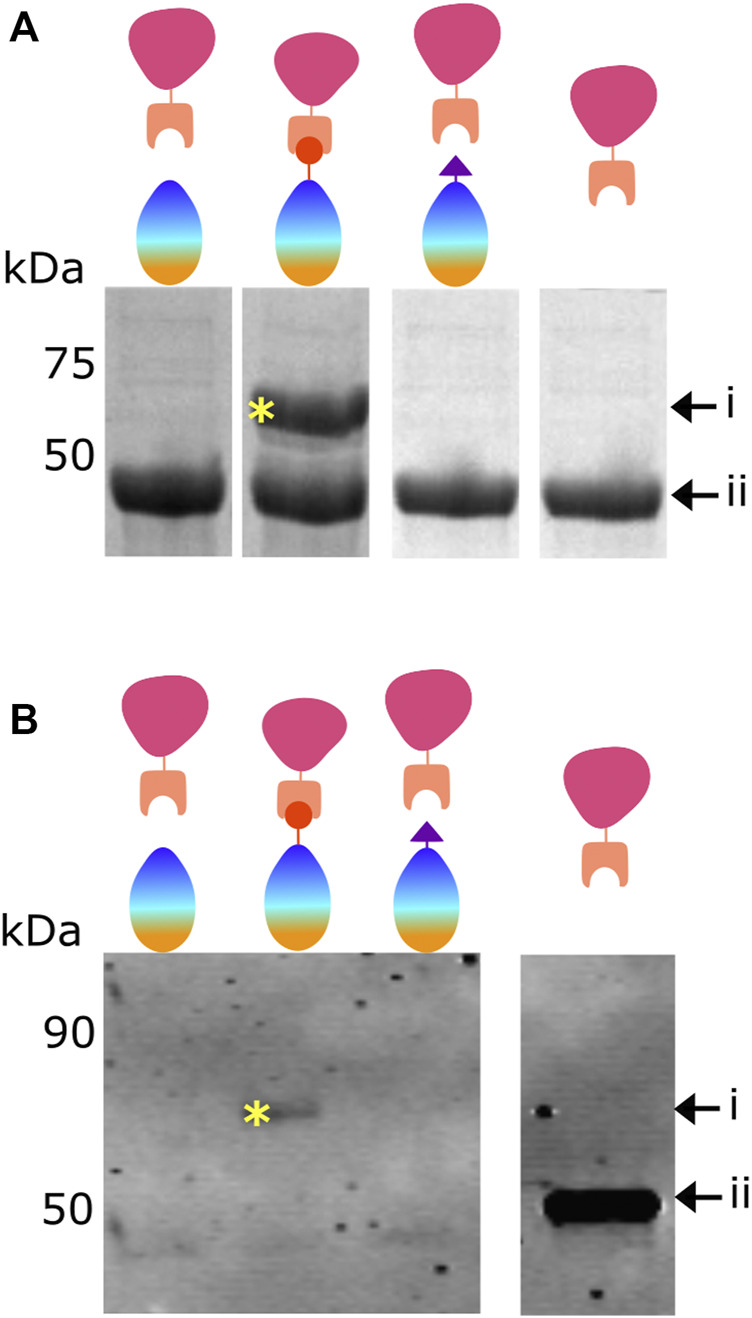
Proteins bind to the BslA surface via a covalent bond. **(A)** Demonstration of covalent bond formation in solution. mCherry-SnC (44.8 kDa, labelled as ii) was incubated with BslA-WT, BslA-SnT, or BslA-SpT in solution and the products analysed by SDS-PAGE. A new protein band is seen at a molecular weight of approximately 60 kDa (labelled as i and with a yellow asterisk), only in the mCherry-SnC plus BslA-SnT reaction mix. Because it withstands the SDS-PAGE separation, the new band represents the covalently linked mCherry-SnC + BslA-SnT. Because mCherry-SnC protein is present in excess, bands corresponding to unreacted BslA proteins are not shown here for simplicity. The complete, uncropped SDS-PAGE gel is shown in Supplementary Figure S5. **(B)** Demonstration of covalent bond formation to a surface. Surfaces of BslA-WT, BslA-SnT, or BslA-SpT on a hydrophobic glass surface were reacted with mCherry-SnC. Excess protein was removed and the surface gently washed. The surfaces were then vigorously washed with SDS loading buffer, to extract the BslA surface and any associated proteins, and the samples analysed by SDS-PAGE. Because the amounts of protein were low, a Western blot was performed, and the membrane probed with an anti-mCherry primary antibody. A band at approximately 60 kDa is evident only with the BslA-SnT plus mCherry-SnC, labelled with a yellow asterisk. Because it withstands the SDS-PAGE separation, we interpret the new band as representing the covalently linked mCherry-SnC + Bsla-SnT. Uncropped versions of both SDS-PAGE gel and Western blot are supplied in Supplementary Figure S5.

Figure 2A shows purified mCherry-SnC added to BslA-WT, BslA-SnT or BslA-SpT in solution. After incubation, the mixture was analysed by SDS-PAGE. The presence of a new band corresponding to the mCherry-SnC-BslA-SnT fusion is evident only when the cognate pair are mixed, indicating specific and covalent attachment of mCherry-SnC to BslA-SnT.

We next sought to determine if mCherry-SnC is covalently linked to BslA-SnT after reaction with the BslA surface. To this end, we incubated BslA-WT, BslA-SnT, and BslA-SpT surfaces with mCherry-SnC. After washing, we extracted all proteins from the surface using SDS sample buffer and analysed the mixtures by SDS-PAGE. Because the amounts of the materials are low, we performed a Western blot, probing the gel with anti-mCherry antibodies. A band corresponding to a covalently joined complex is only present in the lane corresponding to mCherry-SnC + BslA-SnT.

This result supports the conclusion that BslA surfaces can be covalently functionalised with a protein of interest using the Tag/Catcher technology.

### BslA Proteins do not Diffuse Laterally in the Surface Coating

Having established covalent attachment of fluorescent proteins, we sought to investigate the lateral mobility of BslA molecules in the surface. Such knowledge is important because it underlies the feasibility of localised attachment of different proteins to different areas of a BslA surface. We investigated BslA lateral mobility in the surface using Fluorescence Recovery After Photobleaching (FRAP).

**FIGURE 3:**
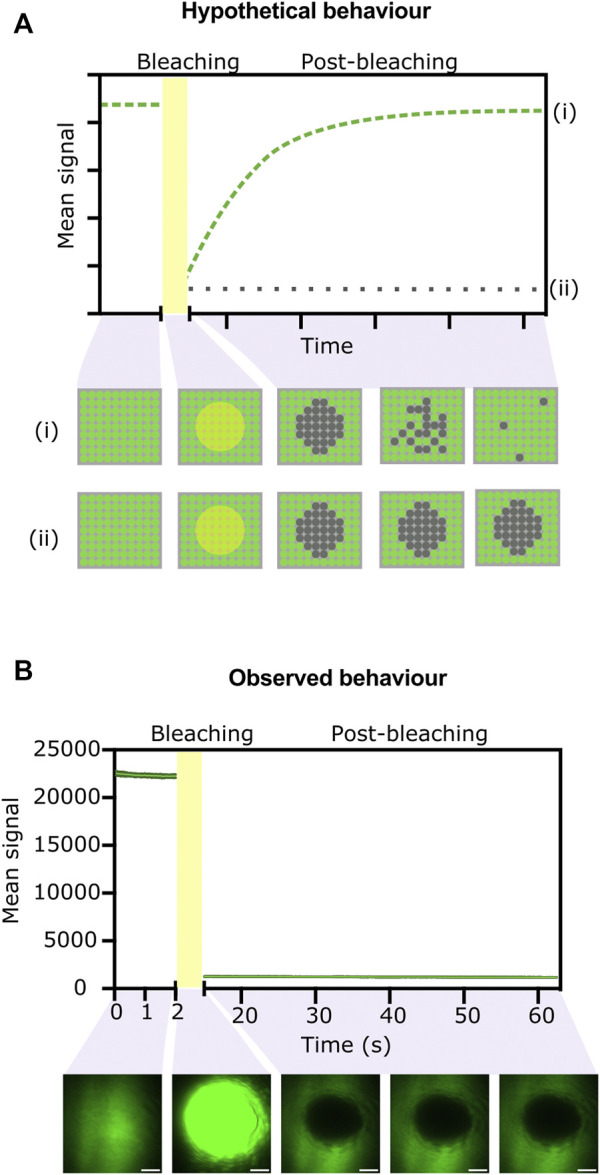
FRAP experiments indicate that proteins do not move laterally in the BslA surface. **(A)** Hypothetical data showing the two extreme possibilities after a FRAP experiment. If the surface exhibits significant lateral mobility, the fluorescence in a bleached area will be recovered over time as non-bleached molecules move in. The expected data from this scenario is indicated by the dashed green line (i) on the graph. The behaviour is illustrated in cartoon form in the panel below, which shows a top-down view of the proteins on the surface. Conversely, if the fluorescent molecules do not diffuse laterally, fluorescence will not be recovered after photobleaching (dotted grey line on the graph, (ii) and matching cartoon). **(B)** Experimentally observed data for a BslA-SpT-SpC-GFP surface. No recovery of fluorescence after photobleaching is observed, indicative of no lateral movement. Error bars show standard deviation, but they are not clearly visible because the variability between replicates was very small (*n* = 4). Representative images of the surface at different stages of the experiment are shown below the graph. Images have been colored green using Fiji. Scale bar represents 10 µm.

We hypothesised that if the BslA-GFP fusions in the surface can move laterally, we would see recovery of fluorescence within a photobleached area of the surface. Conversely, if the BslA proteins do not move laterally in the surface, after photobleaching of a given area we would expect to see no recovery of fluorescence. A schematic illustration of these two scenarios is shown in (Figure 3A).

Figure 3B shows the experimentally observed behaviour. We observe no recovery after photo-bleaching an area of the BslA-SpT-SpC-GFP surface. This behaviour indicates that the BslA proteins do not diffuse laterally in the surface. This experiment was also repeated over longer time scales, and no recovery of fluorescence in the bleached area was observed even after 30 min (Supplementary Figure S4).

We therefore conclude that BslA proteins in a surface do not diffuse laterally. This result is important because it suggests that localised application of different Catcher-proteins to different areas of a BslA-Tag surface should be feasible.

The data shown here also supports our hypothesis that the fluorescent proteins are covalently attached to the BslA surface. If the interaction were non-covalent one might expect to observe the diffusion of fluorescent proteins back into the photobleached area as they unbind and rebind to the BslA surface.

### BslA Surfaces can Capture Proteins Made by CFPS, *In Situ*

**FIGURE 4:**
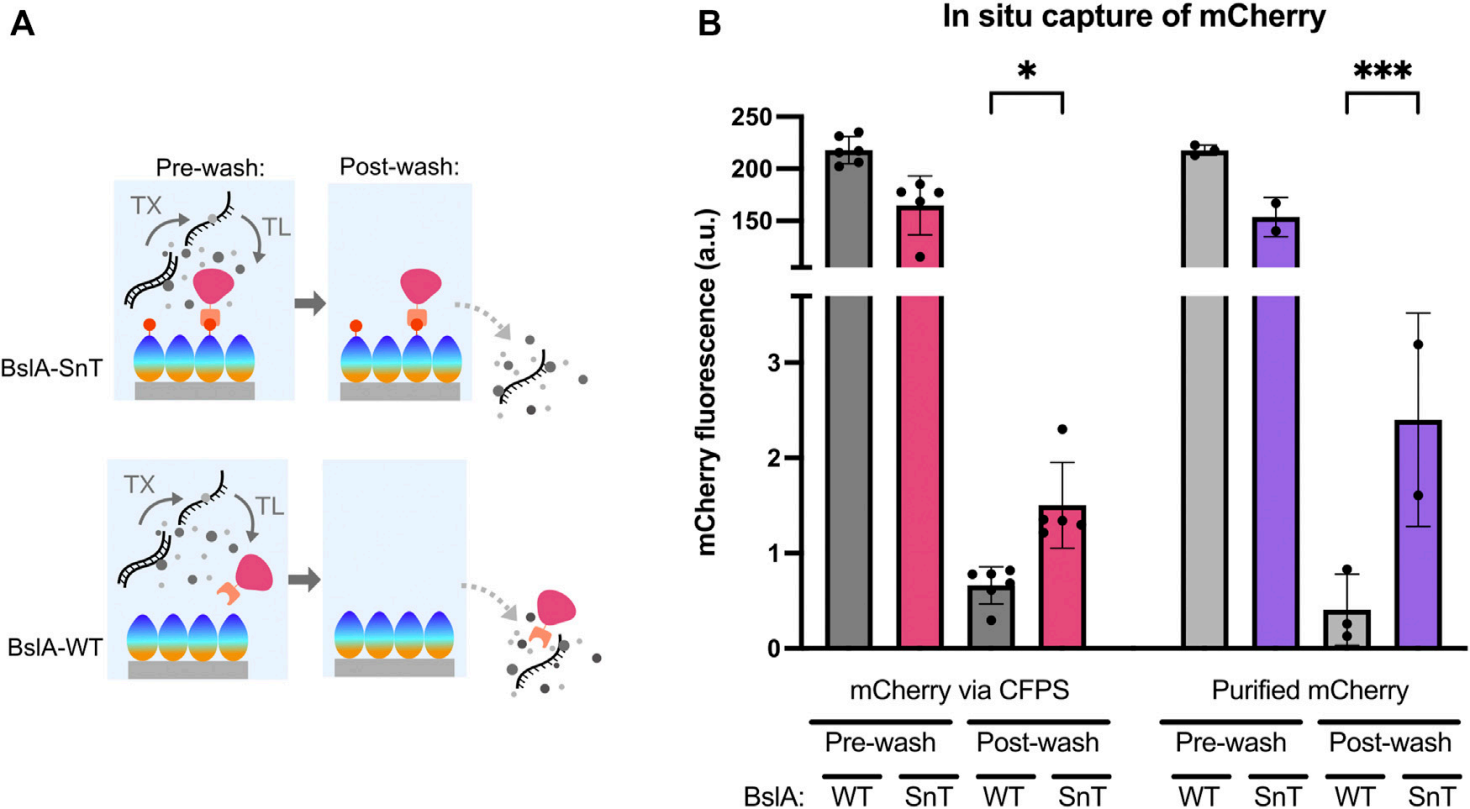
BslA surfaces specifically capture protein produced in a cell-free reaction. **(A)** Schematic illustration of the experiment: A BslA-SnT or BslA-WT surface is assembled on the surface of a hydrophobically coated glass slide. mCherry-SnC is produced by CFPS above this surface. Because an equal amount of DNA is added to each reaction, we expect approximately equal amounts of fluorescent protein will be produced in both wells. After CFPS, the wells are washed. Only mCherry-SnC that is bound to the surface will be retained in the well after washing. **(B)** Experimental data resulting from the experiments illustrated in (A). A similar amount of total fluorescence is observed in the CFPS reactions performed above the BslA-WT and BslA-SnT surfaces. The wells are then washed, and the retained mCherry fluorescence is measured. Higher retention of fluorescence is observed for the CFPS performed above a BslA-SnT surface than above a BslA-WT surface, indicating capture of the mCherry protein synthesised *in situ*. When purified mCherry-SnC protein is added exogenously to the CFPS reaction mixture, rather than synthesised *in situ*, a similar result is obtained: with higher retention of fluorescence in BslA-SnT wells than in BslA-WT wells. Importantly, the fluorescence values of each well before and after washing does not correlate to efficiency of binding, as we expect this concentration of mCherry-SnC to be in excess of the possible binding sites on the surface of each well. Supplementary Information **(C)** provides estimates of maximum binding capacity of the given surface area. Post-wash signal was analysed for significance by one-way ANOVA using Šídák’s multiple comparisons test. Significance is denoted by asterisk on the graph between fluorescence for WT and SnT surfaces post-wash, where * denotes *p* ≤ 0.05 and *** denotes *p* ≤ 0.001. Error bars show standard deviation.

We investigated the ability of self-assembled BslA surfaces to capture proteins that are made in a CFPS reaction. Figure 4A shows a schematic of the experiment, where mCherry-SnC is produced by CFPS above a BslA-SnT or BslA-WT surface. When mCherry-SnC is synthesised, we expect that it will form a covalent bond with a BslA-SnT surface, but not with a BslA-WT surface. Therefore, after removing CFPS constituents and washing the wells, we expect to see mCherry signal retained only in those wells coated with BslA-SnT.

In Figure 4B, we show the relative amounts of mCherry-SnC produced in the CFPS reactions. Post washing, mCherry signal is only retained in the wells coated with a BslA-SnT surface. The 4 bars on the right-hand side of Figure 4B visualise data where purified mCherry-SnC protein (5 µM) is added to a CFPS reaction with no DNA for protein synthesis. These samples serve as a control, and the results indicate that protein synthesised in CFPS and protein added exogenously to the CFPS mixture can bind to the surface specifically and with a similar efficiency.

The data we present here indicates not only the ability of the BslA surfaces to capture proteins produced by CFPS, but also their robustness over a 12 h incubation at 37°C during the CFPS reaction.

## Discussion

CFPS is a powerful approach with many applications. Here we demonstrate how CFPS can be expanded to include a new method of surface capture.

We demonstrate that the BslA protein spontaneously assembles into a surface, and that CFPS can be performed over such a surface. The method we present can be applied either to capture a protein produced in the cell-free reaction, or to bind purified protein. Importantly, the attachment of the protein to the BslA surface is covalent, and therefore robust to washing and other perturbations (Supplementary Figure S3). Binding proteins to the BslA surface can have differing background levels depending on the protein used and should be tested on a case-by-case basis if this is important for the desired application. To further reduce non-specific interactions between the target protein and BslA surface, washing conditions can be further optimised to ensure all proteins are covalently bound to the surface and not non-specifically stuck.

Our demonstration, by FRAP, that the BslA proteins do not move laterally on the hydrophobic surface suggests that it will also be possible to use this method to immobilise different proteins in different areas of the surface.

We envision that surface capture of a protein produced by CFPS could be used either to allow multiple rounds of screening of the activity of that protein against different substrates or to allow for consecutive rounds of CFPS to occur above the same surface. Also, it is a means by which an exogenously added protein could be present on the surface during a CFPS reaction.

The method we describe represents a simple yet powerful method to create functionalised surfaces that are compatible with CFPS. The surfaces are robust over the duration of a CFPS reaction, and the desired protein can be specifically captured from a heterogeneous mixture. By using proteins that are genetically encodable, and which can be fused to any protein of interest, at a unique position, this method is both cost-effective and versatile.

## Materials and Methods

### *E. coli* General Methods

Standard overnight cell growth: *E. coli* cells were picked from a single colony on an LB agar plate with the appropriate antibiotic into 5 ml LB (10 g/L peptone, 5 g/L NaCl, 5 g/L yeast extract) with the appropriate antibiotic and grown overnight at 37°C with shaking (220 rpm). For DNA preparation, TOP10 cells were used [F- *mcrA* Δ(*mrr*-*hsd*RMS-*mcr*BC) φ80*lacZ*ΔM15 Δ*lacX*74 *nup*G *recA*1 *araD*139 Δ(*ara-leu*)7697 *gal*E15 *gal*K16 *rps*L(StrR) *end*A1 λ-]. For protein expression, BL21 Gold (DE3) cells were used [F- *omp*T *hsdSB*(*rB*–*mB*–) *dcm* (TetR) *gal* λ(DE3) *endA* The]. For CFPS lysate production, Rosetta-gami2 cells were used [Δ(ara-leu)7697 ΔlacX74 ΔphoA PvuII phoR araD139 ahpC gale galK rpsL F’[lac + lacIq pro] gor522:Tn10 trxB pRARE2 (Cam^R^, Str^R^, Tet^R^)]. Plasmids were transformed into competent *E. coli* cells following standard protocols (Green and Sambrook, 2012).

DNA purification and quantification: Plasmids were purified from *E. coli* following protocols described by the manufacturer using the QIAprep^®^ Spin Miniprep Kit (Qiagen). Linear DNA was purified from PCR mixture or agarose gels using the Promega Wizard^®^ SV Gel and PCR Clean-up System following protocols described by the manufacturer. Purified DNA solutions were quantified by A260 and stored at −20°C.

DNA sequencing: Plasmid sequences were verified by DNA sequencing performed either by DNA Sequencing & Services (www.dnaseq.co.uk) or Source Bioscience (www.sourcebioscience.com), using primers provided by the company.

### Protein Expression and Purification

Cell growth and protein expression: Overnight cultures were diluted 100- fold into LB containing the appropriate antibiotic and grown at 37°C with shaking until OD600 reached 0.6–0.8. Protein expression was induced by addition of isopropyl β-d-1-thiogalactopyranoside (IPTG) to a final concentration of 1 mM and growth continued for a further 20 h at 20°C with shaking. Cells were collected by centrifugation at 6,000 × *g* for 10 min and pellets stored at −20°C until needed.

Lysis and clarification: Cells were resuspended in lysis buffer (Supplementary Table S1) containing cOmplete Protease Inhibitor Cocktail (Sigma-Aldrich) according to manufacturer’s instructions at a ratio of 1:50 volume of buffer to original cell culture volume. Resuspended cells were sonicated (Soniprep 150, MSE) on ice for 30 s, followed by a 30 s rest period. This sonication-rest cycle was repeated until cell lysis was achieved. Clarified cell lysates were prepared by centrifugation at 10,000 × *g* for 30 min at 4°C.

Affinity purification: mCherry-SnC and GFP-SpC were purified via hexahistidine tag using Ni-NTA agarose (Qiagen) according to the manufacturer’s instructions. BslA-fusion proteins were purified via GST tag using GST agarose (ThermoFisher) according to the manufacturer’s instructions. The GST tag was cleaved on column with PreScission Protease (GE Healthcare) according to the manufacturer’s instructions. Purification was monitored using SDS-PAGE. Fractions containing protein at approximately 90% or greater purity, were pooled and dialysed against desired the storage buffer.

Note on BslA purification: During purification of BslA proteins, solution containing concentrated and pure protein could often look opaque and white in colour. When this happened, the solutions were left to stand at room temperature to allow for re-solubilisation of BslA protein, which could take between 5—30 min, depending on concentration of solution. Freeze thaw cycles were always kept to a minimum with pure protein solutions, but even more stringently with BslA: pure protein was aliquoted into small volumes before freezing at −20°C. For experiments involving functionalisation of surfaces, BslA solutions were only thawed once. These precautions were developed according to our own observations and in consultation with an author of (Bromley et al., 2015).

Size exclusion chromatography (SEC): Further purification of BslA proteins by size exclusion chromatography (SEC) with a Superdex-75 column was used when affinity chromatography did not provide sufficient purity.

Protein quantification: Protein concentration was determined by measuring absorbance at 280 nm using the extinction coefficient of each protein calculated from the amino acid sequence using the tools available on Benchling [Biology Software]. (2022) Retrieved from https://benchling.com.

Protein concentration: Buffer exchange and simultaneous concentration of protein solutions was performed using Amicon^®^ Ultra Centrifugal Filters (Merck). Columns were selected based on the sample volume and POI size. Generally, the MW cut off was chosen to be at least 1.5 × smaller than that of the POI.

Dialysis: Buffer exchange of protein solutions was performed by dialysis using SnakeSkin Dialysis Tubing (ThermoFisher, various sizes depending on POI) according to manufacturer’s instructions. Typically, samples were dialysed into 1 L buffer overnight at 4°C with stirring, followed by dialysis into 1 L fresh buffer twice more, for a period of 1 h each at 4°C with stirring.

SDS-PAGE: Protein expression, purification and reactivity was monitored using SDS-PAGE. For testing covalent bond formation between SpT/SpC and SnT/SnC fused proteins, each protein was incubated together at approximately equimolar concentrations for 1 hour at room temperature. Samples in 1x SDS loading buffer were heated at 100°C for 10 min before loading on SDS-PAGE gels alongside Precision Plus Protein Dual Xtra Prestained Protein Standards as molecular weight marker. Protein bands were visualised by staining using InstantBlue^®^ Coomassie Protein Stain according to manufacturer’s instructions and were subsequently imaged using a Bio-Rad Gel Doc XR + system with white light filter in Coomassie mode.

Western blot: Protein samples were prepared and loaded onto an SDS-PAGE gel with Chameleon Duo Prestained Protein Ladder as a molecular weight marker. The Western blot procedure described in “Near-Infrared Western Blot Detection Document” (Doc. #988-13627, Li-cor) was followed. Antibodies recognising mCherry (Anti RFP-tag, pAb, Rabbit; A00682, GenScript) were used as a primary antibody at a dilution of 1:3,000. IRDye 680RD Goat anti-Rabbit (925-68071, Li-cor) was used as a secondary antibody at a dilution of 1:20,000. Blotted membranes were visualised using an Odyssey CLx Infrared Imaging System (Li-cor) and analysed by Image Studio Lite software (Li-cor).

### Surface Functionalisation

Preparation of glass slides: Ultra-clean, hydrophobic glass microscope slides were prepared following methods adapted from the literature (Cras et al., 1999). 150 ml HCl was slowly added into 150 ml MeOH in a glass beaker and gently swirled to mix. Slides (Thermo Scientific plain microscope slides, 12332098) were added to the solution and incubated at room temperature for 1 hour. Slides were removed and added to a fresh beaker with 300 ml distilled water, gently swirled to mix and this wash step was repeated an additional four times. Slides were tapped dry (avoiding touching the surface) and dried completely by incubation in a drying oven at 90°C for 2 hours. To add the hydrophobic coating to the cleaned sides, Dichloromethylsilane was dissolved in Trichloroethylene to a final concentration of 0.05% and incubated with the dried glass slides for 1 hour. Glass slides were transferred to a MeOH solution and incubated for 1 min, then slides were removed and incubated again with MeOH three more times. Slides were rinsed twice in distilled water and dried before packaging with lens paper in a sealed container. Slides were still sufficiently clean and hydrophobic for the experiments described after storage for up to 3 months. Hydrophobicity was assessed by measuring the contact angle of a water droplet on the glass surface.

Application of proteins to BslA surfaces: ProPlate Multi-Well Chambers (Grace Bio-Labs) allowed up to four slides with adaptors to be combined in one adaptor plate (ProPlate Multi-Array Slide System (Grace Bio-Labs) and imaged in a plate reader. 10 μL BslA protein (22 μM) was incubated in each well at room temperature for 1 h with high humidity to prevent evaporation. After surface formation, excess BslA was removed by pipette. 10 μL protein of interest was incubated with the well for 1 h to allow the SpyTag/SpyCatcher reaction to occur. Excess protein was removed from each well by pipetting, adaptors were removed and the whole slide was washed with 50 ml ultrapure water. Fluorescence was measured using a FLUOstar Omega plate reader with appropriate filters. For GFP = excitation 485 nm, emission 520 nm, for mCherry = excitation 584 nm, emission 620 nm, with gain set between 1500—2500.

### Cell-Free Protein Synthesis

Lysate production: This protocol is adapted from (Kwon and Jewett, 2015), and was optimised as shown in Supplementary Figure S1 and functionally demonstrated in Supplementary Figure S2. Typically, addition of 5 nM plasmid DNA to our homemade CFPS system yielded 5 μM protein in a 10 μL reaction. 2xYTPG (16 g/L tryptone, 10 g/L yeast extract, 5 g/L NaCl, 7 g/L KH2PO4 3 g/L K2HPO4, 18 g/L glucose) was inoculated with 1/200 dilution of overnight cultures of Rosetta-gami2 *E. coli* cells. Cultures were grown for 2 h at 37°C with shaking, then induced with 0.4 mM IPTG and grown for a further 2 h in the same conditions, before growth arrest by placing on ice. Cells were harvested by centrifugation at 10,000 ×g for 10 min at 4°C, the supernatant was discarded, and cell pellets were resuspended with 80 ml Buffer A (Supplementary Table S1) per 400 ml cells harvested. Cells were collected by centrifugation at 4,500 rpm for 10 min at 4°C. The washing and cell harvesting process was repeated twice more, and cell pellets stored at −80°C for future downstream processing. Cell pellets were resuspended with 1 ml buffer A per 1 g wet cell mass and homogenised by vortexing. 1.5 ml aliquots were taken from the total mixture and sonicated (Fisher120 W sonicator with probe for 0.5–15 ml) with pulses of 10 s on, and 10 s off, until a total energy output of 556 J was achieved, while incubated on ice. Lysate was clarified by centrifugation at 12,000 *× g* at 4°C for 10 min. Supernatant was removed and re-spun under the same conditions. Clarified supernatant was placed in a clean 1.5 ml Eppendorf tube and incubated at 37°C for 1.5 h with shaking (220 rpm) in a “run-off” reaction (the impact of both run-off and dialysis on functionality of CFPS lysate is detailed further in Supplementary Figure S1). Samples were then centrifuged at 12,000 × g at 4°C for 10 min. Supernatant was removed and aliquoted into 25 μL samples, which were stored at −80°C until they were required for use.

Energy solution production: Energy solution was assembled from stock solutions of all constituents. Amino acid stock solution was made of final concentration 50 mM each of the following amino acids: Alanine, Arginine, Asparagine, Aspartate, Cysteine, Glutamate, Glutamine, Glycine, Histidine, Isoleucine, Leucine, Lysine, Methionine, Phenylalanine, Proline, Serine, Threonine, Tryptophan, Valine. Tyrosine was prepared separately, in an acidic solution (pH ∼5.2) also at a final concentration of 50 mM. Stock batches of energy solution were prepared in volumes of 3 ml, with the following recipe: HEPES (pH 8) 200 mM, ATP 6 mM, GTP 6 mM, CTP 3.6 mM, UTP 3.6 mM, tRNA 0.8 mM, CoA 1.04 mM, NAD 1.32 mM, cAMP 3 mM, Folinic acid 0.27 mM, Spermidine 4 mM, 3-PGA 120 mM, Amino acids 6 mM, Tyrosine 3 mM, PEG-8000 8%, Mg-glutamate 42 mM, K-glutamate 400 mM, DTT 1 mM.

Assembly of cell-free reaction: Cell free reactions were prepared with a final volume of 10 μL in wells of a 384 microplate (Greiner, 781906). Energy solution, lysate, DNA, and buffer A were combined in a 1:1:1:1 ratio by volume in each well. When required, master mixes were prepared which contained DNA, energy solution and buffer before dispensing into appropriate wells. Lysate was always added to each well individually and pipetted onto the side of the well wall. This protocol provided a useful visual note of progress through the plate, but also allowed for the initiation of the cell-free reaction at the same time in every well, by centrifuging the plate at 500 rpm for 2 min at 4°C. This mixed all components simultaneously and removed any air bubbles from pipetting. After centrifugation, 35 μL BioRad chillout wax was added to each well to prevent evaporation of samples. Plates were sealed with non-breathable film and placed in the plate reader.

Preparation of DNA for cell-free reactions: Plasmid DNA was used for CFPS reactions, with expression from a pTrc promoter upstream of protein coding DNA sequence (full sequences available in Supplementary Data (D)). DNA used in cell-free reactions required a higher level of purity than those for standard molecular biology procedures. DNA was extracted from cells using the PureLink HiPure Plasmid Maxiprep Kit (Invitrogen) following described protocols. DNA was further purified by DNA Clean & Concentrator Kit (Zymo Research) following described protocols. Final DNA concentration was measured by absorbance at 260 nm on a Nanodrop (DeNovix DS-11) and DNA was stored at −20°C until required.

### FRAP Microscopy

Samples for FRAP experiments were prepared in triplicate according to methods previously described, on glass coverslips with CultureWell Reusable Gaskets. BslA-WT or BslA-SpT was used at a concentration of 11 μM, and GFP-SpC at a concentration of 5 μM.

After incubation of proteins with the surface, the gasket was removed, and the coverslip washed thoroughly with ddH2O. Samples were then imaged using a custom-built TIRF (Total Internal Reflection Fluorescence) microscope (as previously described in (Pérez-Pi et al., 2019), using a 488 nm laser to excite and illuminate the GFP molecules throughout imaging. Photobleaching of one section of the surface was achieved using 405 nm laser excitation. Experiments were typically set up to collect data for 2 s pre-bleach, bleach for 10 s, and then collect data for the 30 s following. For some experiments, data was collected post-bleach for 5 min. The microscope was set up for each experiment as following. Laser light (Colbalt Diode Laser Systems, Cobalt, Sweden) were aligned and directed parallel to the optical axis at the edge of a 1.49 NA TIRF objective (CFI Apochromat TIRF 60XC Oil, Nikon, Japan), mounted on an inverted Nikon TI2 microscope (Nikon, Japan). The fluorescence was separated from the returning TIR beam by a dichroic mirror Di01-R405/488/561/635 (Semrock, Rochester, NY, United States), passed through the appropriate filters (Semrock, NY, United States) and then recorded on an EMCCD camera (Delta Evolve 512, Photometrics, Tuscon, AZ, United States). Each pixel was 103 nm in length. Data was analysed with Python code using Fiji to track mean fluorescence over time from the centre of the photobleached area. This numerical data was visualised in GraphPad Prism v8.1.2. To prepare the images shown in Figure 3, the raw data were processed using Fiji (Schindelin et al., 2012). The images were false coloured green by converting the greyscale image to RGB and splitting the image to only show the green channel.

## Data Availability

The original contributions presented in this study are included in the Supplementary Material. Further inquires can be directed to the corresponding authors.
