# Supplementary material for: Self-Assembling Protein Surfaces for In Situ Capture of Cell-Free-Synthesized Proteins
Source: Front Bioeng Biotechnol. 2022 Jul 7;10:915035. doi: 10.3389/fbioe.2022.915035 (PMC9300835; doi:10.3389/fbioe.2022.915035)
Supplement: Supplementary file 1 [file DataSheet1.docx]

**Supplementary Material**

**Self-assembling protein monolayers for in situ capture of cell-free-synthesized proteins**

Thornton *et al*.

**Contents**

1. Supplementary figures:

Figure S1. Lysate production optimisation.

Figure S2. DNA concentration titration in our homemade CFPS system.

Figure S3. Washing BslA surfaces with additives does not perturb the monolayer.

Figure S4. Monitoring fluorescence recovery for 30 minutes with FRAP BslA-GFP experiments.

Figure S5. Uncropped gel images relating to Figure 2.

1. Supplementary Table S1: Buffers used in this study.
2. Supplementary information
3. Plasmids used in this study: DNA and protein sequences
4. Supplementary data: provided as a separate excel file
5. **Supplementary figures**


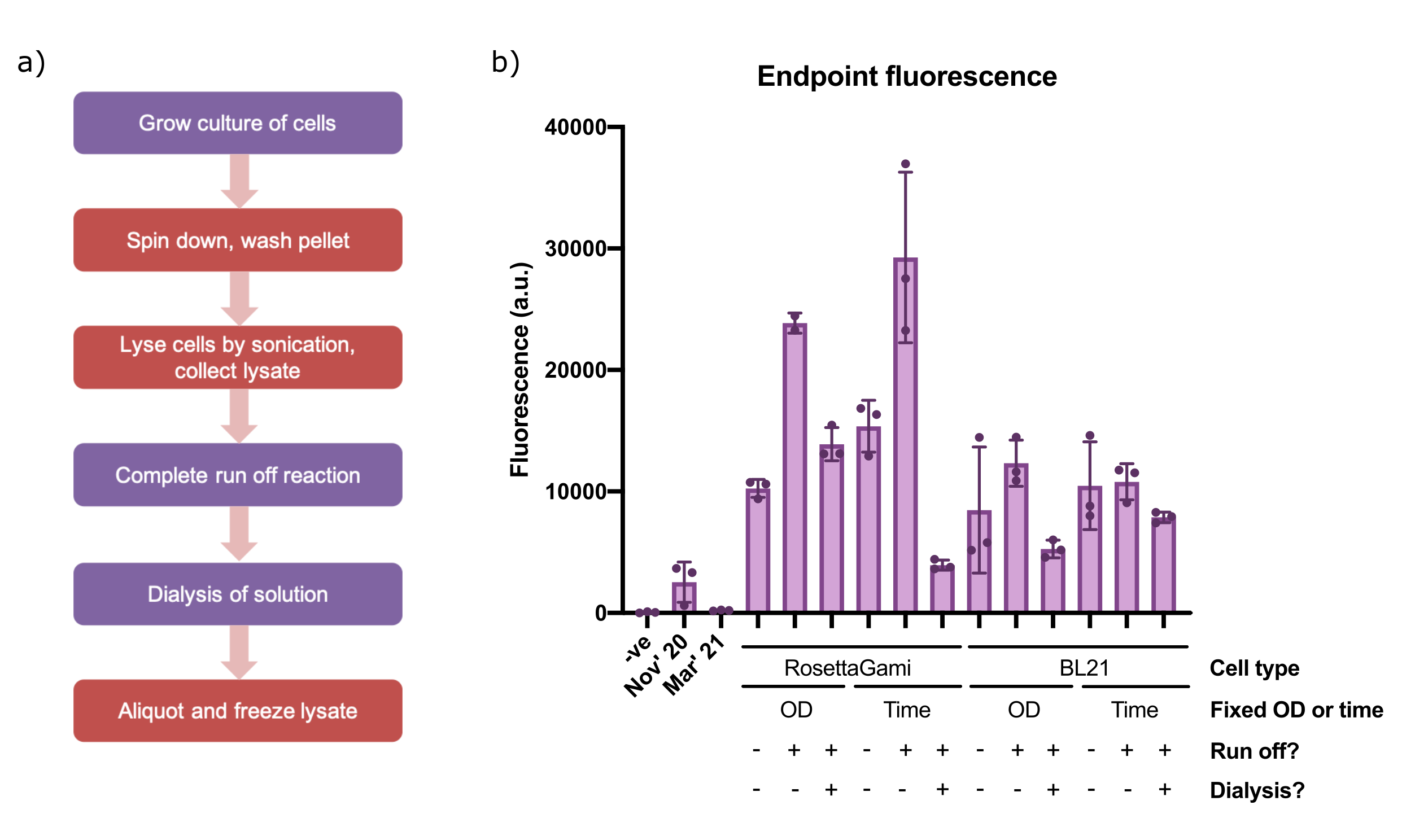


**Figure S1. Optimisation of lysate production for cell-free reactions.** (**a**) Key steps in the method for lysate preparation. In purple are the steps we explore variations of in these experiments. In red are the steps kept constant throughout experiments. (**b**) Endpoint fluorescence for mCherry production with different preps of lysates. CFPS reactions supplemented with 2.5 nM DNA and incubated for 12 hours at 37°C. Negative control (no DNA added) is shown as the first bar in purple (named -ve). Lysates produced using old protocol are annotated as Nov’ 20 and Mar’ 21, referring to the month they were made. Conditions used for the preparation of each lysate are shown in the table below the x axis. Fluorescence measured at 584 nm excitation and 610 nm emission, gain = 2000. Data is blank subtracted. Error bars, s.d. (n=3).

**Figure S2. DNA concentration titration in our homemade CFPS system.** Red fluorescence corresponding to mCherry synthesis increases in a linear fashion as DNA added to the reaction increases until 5 nM. From 5 to 6.7 nM DNA final concentration, there is not a significant increase of protein expression, indicating 5 nM is sufficient for high protein expression in our CFPS system. Therefore, 5 nM was used as a standard plasmid concentration for all CFPS experiments. From calibration with purified mCherry protein at known concentrations, we estimate a final concentration of 5 nM plasmid DNA in a 10 µL CFPS reaction will yield a final protein concentration of approximately 5 µM in a standard reaction using our homemade lysate and energy solution. A standard CFPS reaction corresponds to 37°C incubation for 12 hours. Fluorescence measured at 584 nm excitation and 610 nm emission, gain = 2000. Data is blank subtracted. Error bars, s.d. (n=3).

**Figure S3. Washing BslA surfaces with additives does not perturb the monolayer**. BslA surfaces were formed as described, and mCherry-SnC incubated with both SnT and WT BslA surfaces. After washing with water, five further washes were tested on five different sets of surfaces and fluorescence compared. Retention of red fluorescence in the SnT surfaces after washing implies the BslA monolayer and covalently linked mCherry protein has not been washed away.

**Figure S4. Monitoring fluorescence recovery after FRAP for 30 minutes.** After photobleaching of GFP proteins bound to the BslA monolayer, fluorescence was monitored over 30 minutes. Fluorescence is not recovered within this time. In these experiments, a lower laser power was used for bleaching, and a higher-powered laser for general fluorescence monitoring than in the data shown in Figure 3. This is the reason for the difference in the appearance of the data. Importantly, no recovery of fluorescence is seen over 30 minutes after photobleaching. Error bars are shown, but not clearly visible due to high similarity of replicates (n=2).


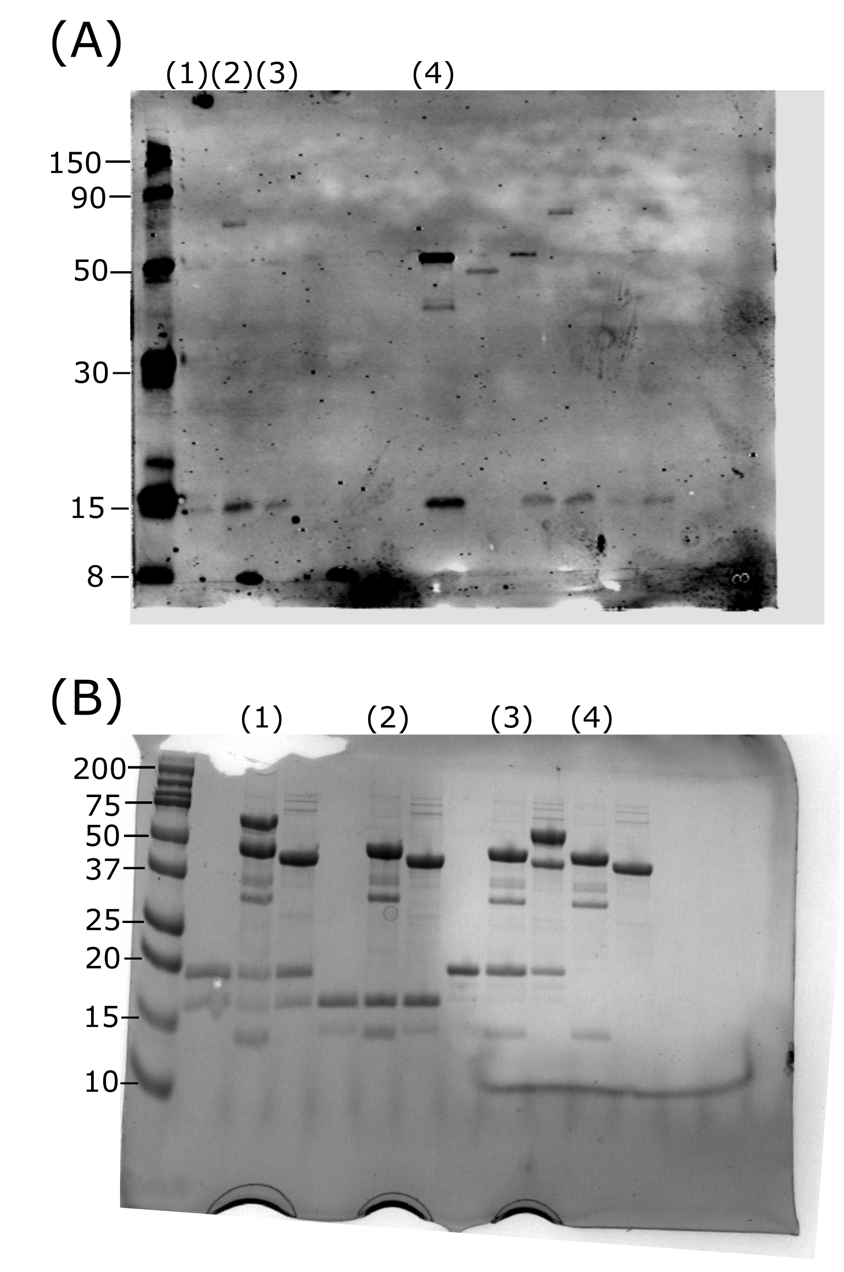


**Figure S5. Uncropped gel images for Figure 2.** (**A**) Western blot probed with anti-mCherry antibody. The first lane contains a molecular weight marker and band sizes are denoted to the left-hand side in kDa. Lanes relevant to Figure 2 are numbered and show the following samples. A1: BslA-WT surface incubated with mCherry-SnC. A2: BslA-SnT surface incubated with mCherry-SnC. A3: BslA-SpT surface incubated with mCherry-SnC. A4: mCherry-SnC in solution. (**B**) SDS-PAGE gel stained with Coomassie. The first lane contains a molecular weight marker and corresponding sizes are denoted to the left-hand side in kDa. Lanes relevant to the experiments shown in Figure 2 are numbered and show the following. B1: BslA-SnT incubated with mCherry-SnC in solution. B2: BslA-WT incubated with mCherry-SnC in solution. B3: BslA-SpT incubated with mCherry-SnC in solution. B4: mCherry-SnC in solution. Additional bands present in the lanes of this protein gel are likely to be degraded versions of mCherry-SnC protein, which could not be separated from the purified protein sample.

1. **Supplementary Table**

**Supplementary Table S1. Buffers used in this study.** Buffers were used for a range of biochemical techniques, and the recipe for all references in this paper methods are listed here. When sterile buffers were required, they were either autoclaved or passed through a 0.22 µm filter.

| **Name** | **Ingredients** | **Purpose** | **Source** |
| --- | --- | --- | --- |
| Lysis Buffer for His purification | 50 mM NaH_2_PO_4_  300 mM NaCl  10 mM imidazole (optional) | Purification of proteins via His-tag | QIAexpressionist manual |
| Wash buffer for His purification | 50 mM NaH_2_PO_4_  300 mM NaCl  20 mM  imidazole | Purification of proteins via His-tag | QIAexpressionist manual |
| Elution buffer for His purification | 50 mM NaH_2_PO_4_  300 mM NaCl  250 mM imidazole | Purification of proteins via His-tag | QIAexpressionist manual |
| Equilibration/Wash Buffer for GST purification | 50 Mm Tris-HCl  150 mM NaCl  pH 8.0 | Purification of proteins via GST-tag. | GST Agarose manual |
| Elution Buffer for GST purification | 50 mM Tris  150 Mm NaCl  10 mM reduced glutathione  pH 8.0 | Purification of proteins via GST-tag | GST Agarose manual |
| Regeneration buffer #1 for GST resin | - 1. M Tris   0.5 M NaCl  0.1% SDS  pH 8.5 | Purification of proteins via GST-tag | GST Agarose manual |
| Regeneration buffer #2 for GST resin | - 1. M Sodium acetate   0.5 M NaCl  0.1% SDS  pH 4.5 | Purification of proteins via GST-tag | GST Agarose manual |
| Protease cleavage buffer | 50 mM Tris-HCl (pH 7.0)  150 mM NaCl  1 mM EDTA  1 mM DTT | For cleavage of GST tag via PreScission Protease | PreScission Protease manual |
| Fluorescent protein storage buffer (GFP-SpC and mCherry-SnC) | 10 mM Tris-HCl  25 mM NaCl  pH 7.5 | For storage of fluorescent proteins | Standard laboratory protocols |
| BslA storage buffer | 25 mM phosphate buffer  pH 7.0 | Storage of purified BslA | (Bromley et al., 2015) |
| Buffer A for cell-free | 10 mM tris acetate (pH 8.2)  14 mM magnesium glutamate  60 mM potassium glutamate | Cell-free reactions | (Kwon & Jewett, 2015) |

1. **Supplementary information**

Area-based calculations suggest that approximately 5 x 10^11^ molecules of BslA protein can be bound on the bottom surface of each glass well used in these experiments. This number is consistent with that which we estimate from a comparison of the fluorescence of a well with the maximal amount of SnC-mCherry bound, and serial dilutions of SnC-mCherry in solution (3 x 10^11^).

1. **Plasmids used in this study**

Each plasmid is detailed in the following pages, with both DNA coding sequence and protein amino acid sequence annotated with colour for clarity. All proteins are expressed via the pTac or pTrc promoter. Plasmids are used to express the following proteins:

1. BslA-WT
2. BslA-SpyTag
3. BslA-SnoopTag
4. GFP-SpyCatcher
5. mCherry_SnC

| **Name** | BslA_WT | **Source** | (Williams et al., 2018) |
| --- | --- | --- | --- |
| **Resistance** | Ampicillin | **Total plasmid size** | 5416 |
| **Parent Vector** | pGEX-6P-1 | **Seq Primers** | pGEX fwd + pGEX rev |
| **Benchling link** | https://benchling.com/s/seq-NZ6ufJB836Sq3JUe56E2?m=slm-h97r8cszfw7iz2QVSXsW | | |

**Description**

Wild-Type BslA for monolayer formation. Cleavable GST tag.

**Shading Key**

Tac promoter

LacO

GST-3C-BslA

**DNA**

CTGGCAAATATTCTGAAATGAGCTGTTGACAATTAATCATCGGCTCGTATAATGTGTGGAATTGTGAGCGGATAACAATTTCACACAGGAAACAGTATTCATGTCCCCTATACTAGGTTATTGGAAAATTAAGGGCCTTGTGCAACCCACTCGACTTCTTTTGGAATATCTTGAAGAAAAATATGAAGAGCATTTGTATGAGCGCGATGAAGGTGATAAATGGCGAAACAAAAAGTTTGAATTGGGTTTGGAGTTTCCCAATCTTCCTTATTATATTGATGGTGATGTTAAATTAACACAGTCTATGGCCATCATACGTTATATAGCTGACAAGCACAACATGTTGGGTGGTTGTCCAAAAGAGCGTGCAGAGATTTCAATGCTTGAAGGAGCGGTTTTGGATATTAGATACGGTGTTTCGAGAATTGCATATAGTAAAGACTTTGAAACTCTCAAAGTTGATTTTCTTAGCAAGCTACCTGAAATGCTGAAAATGTTCGAAGATCGTTTATGTCATAAAACATATTTAAATGGTGATCATGTAACCCATCCTGACTTCATGTTGTATGACGCTCTTGATGTTGTTTTATACATGGACCCAATGTGCCTGGATGCGTTCCCAAAATTAGTTTGTTTTAAAAAACGTATTGAAGCTATCCCACAAATTGATAAGTACTTGAAATCCAGCAAGTATATAGCATGGCCTTTGCAGGGCTGGCAAGCCACGTTTGGTGGTGGCGACCATCCTCCAAAATCGGATCTGGAAGTTCTGTTCCAGGGGCCCCTGGGATCCATGGCTGAATCTACATCAACTAAAGCTCATACTGAATCCACTATGAGAACACAGTCTACAGCTTCATTGTTCGCAACAATCACTGGCGCCAGCAAAACGGAATGGTCTTTCTCAGATATCGAATTGACTTACCGTCCAAACACGCTTCTCAGCCTTGGCGTTATGGAGTTTACATTGCCAAGCGGATTTACTGCAAACACGAAAGACACATTGAACGGAAATGCCTTGCGTACAACACAGATCCTCAATAACGGGAAAACAGTAAGAGTTCCTTTGGCACTTGATTTGTTAGGAGCTGGCGAATTCAAATTAAAACTGAATAACAAAACACTTCCTGCCGCTGGTACATATACTTTCCGTGCGGAGAATAAATCATTAAGCATCGGAAATAAATTTTACGCAGAAGCCAGCATTGACGTGGCTAAGCGCAGCACTCCTCCGACTCAGTAACTCGAGCGGCCGCATCGTGACTGACTGACGATCTGCCTCGCGCGTTTCGGTGATGACGGTGAAAACCTCTGACACATGCAGCTCCCGGAGACGGTCA

**Expression Product Sequence**

MSPILGYWKIKGLVQPTRLLLEYLEEKYEEHLYERDEGDKWRNKKFELGLEFPNLPYYIDGDVKLTQSMAIIRYIADKHNMLGGCPKERAEISMLEGAVLDIRYGVSRIAYSKDFETLKVDFLSKLPEMLKMFEDRLCHKTYLNGDHVTHPDFMLYDALDVVLYMDPMCLDAFPKLVCFKKRIEAIPQIDKYLKSSKYIAWPLQGWQATFGGGDHPPKSDLEVLFQGPLGSMAESTSTKAHTESTMRTQSTASLFATITGASKTEWSFSDIELTYRPNTLLSLGVMEFTLPSGFTANTKDTLNGNALRTTQILNNGKTVRVPLALDLLGAGEFKLKLNNKTLPAAGTYTFRAENKSLSIGNKFYAEASIDVAKRSTPPTQ*

| **Name** | BslA_SpyTag | **Source** | (Williams et al., 2018) |
| --- | --- | --- | --- |
| **Resistance** | Ampicillin | **Total plasmid size** | 5473 bp |
| **Parent Vector** | pGEX-6P-1 | **Seq Primers** | pGEX fwd + pGEX rev |
| **Benchling link** | https://benchling.com/s/seq-p5vbF2jAyCHbT7sk33Rc?m=slm-ed9NvmArFXrd2xLCsRUQ | | |

**Description**

For monolayer formation and subsequent functionalization via SpyCatcher. Cleavable GST tag.

**Shading Key**

Tac promoter

LacO

GST-3C-BslA-SpyTag

**DNA**

CTGGCAAATATTCTGAAATGAGCTGTTGACAATTAATCATCGGCTCGTATAATGTGTGGAATTGTGAGCGGATAACAATTTCACACAGGAAACAGTATTCATGTCCCCTATACTAGGTTATTGGAAAATTAAGGGCCTTGTGCAACCCACTCGACTTCTTTTGGAATATCTTGAAGAAAAATATGAAGAGCATTTGTATGAGCGCGATGAAGGTGATAAATGGCGAAACAAAAAGTTTGAATTGGGTTTGGAGTTTCCCAATCTTCCTTATTATATTGATGGTGATGTTAAATTAACACAGTCTATGGCCATCATACGTTATATAGCTGACAAGCACAACATGTTGGGTGGTTGTCCAAAAGAGCGTGCAGAGATTTCAATGCTTGAAGGAGCGGTTTTGGATATTAGATACGGTGTTTCGAGAATTGCATATAGTAAAGACTTTGAAACTCTCAAAGTTGATTTTCTTAGCAAGCTACCTGAAATGCTGAAAATGTTCGAAGATCGTTTATGTCATAAAACATATTTAAATGGTGATCATGTAACCCATCCTGACTTCATGTTGTATGACGCTCTTGATGTTGTTTTATACATGGACCCAATGTGCCTGGATGCGTTCCCAAAATTAGTTTGTTTTAAAAAACGTATTGAAGCTATCCCACAAATTGATAAGTACTTGAAATCCAGCAAGTATATAGCATGGCCTTTGCAGGGCTGGCAAGCCACGTTTGGTGGTGGCGACCATCCTCCAAAATCGGATCTGGAAGTTCTGTTCCAGGGGCCCCTGGGATCCATGGCTGAATCTACATCAACTAAAGCTCATACTGAATCCACTATGAGAACACAGTCTACAGCTTCATTGTTCGCAACAATCACTGGCGCCAGCAAAACGGAATGGTCTTTCTCAGATATCGAATTGACTTACCGTCCAAACACGCTTCTCAGCCTTGGCGTTATGGAGTTTACATTGCCAAGCGGATTTACTGCAAACACGAAAGACACATTGAACGGAAATGCCTTGCGTACAACACAGATCCTCAATAACGGGAAAACAGTAAGAGTTCCTTTGGCACTTGATTTGTTAGGAGCTGGCGAATTCAAATTAAAACTGAATAACAAAACACTTCCTGCCGCTGGTACATATACTTTCCGTGCGGAGAATAAATCATTAAGCATCGGAAATAAATTTTACGCAGAAGCCAGCATTGACGTGGCTAAGCGCAGCACTCCTCCGACTCAGGGCGGCTCCGGTGGTAGCGCGCATATCGTGATGGTCGACGCATACAAACCGACCAAATAACTCGAGCGGCCGCATCGTGACTGACTGACGATCTGCCTCGCGCGTTTCGGTGATGACGGTGAAAACCTCTGACACATGCAGCTCCCGGAGACGGTCA

**Expression Product Sequence**

MSPILGYWKIKGLVQPTRLLLEYLEEKYEEHLYERDEGDKWRNKKFELGLEFPNLPYYIDGDVKLTQSMAIIRYIADKHNMLGGCPKERAEISMLEGAVLDIRYGVSRIAYSKDFETLKVDFLSKLPEMLKMFEDRLCHKTYLNGDHVTHPDFMLYDALDVVLYMDPMCLDAFPKLVCFKKRIEAIPQIDKYLKSSKYIAWPLQGWQATFGGGDHPPKSDLEVLFQGPLGSMAESTSTKAHTESTMRTQSTASLFATITGASKTEWSFSDIELTYRPNTLLSLGVMEFTLPSGFTANTKDTLNGNALRTTQILNNGKTVRVPLALDLLGAGEFKLKLNNKTLPAAGTYTFRAENKSLSIGNKFYAEASIDVAKRSTPPTQGGSGGSAHIVMVDAYKPTK*

| **Name** | BslA_SnoopTag | **Source** | (Williams et al., 2018) |
| --- | --- | --- | --- |
| **Resistance** | Ampicillin | **Total plasmid size** | 5479 bp |
| **Parent Vector** | pGEX-6P-1 | **Seq Primers** | pGEX fwd + pGEX rev |
| **Benchling link** | https://benchling.com/s/seq-Noc2VLUMMRFKovVldmrK?m=slm-OPBKlZPfu8pjSAoNxKj8 | | |

**Description**

For monolayer formation and subsequent functionalization via SnoopCatcher. Cleavable GST tag.

**Shading Key**

Tac promoter

LacO

GST-3C-BslA-SnoopTag

**DNA**

CTGGCAAATATTCTGAAATGAGCTGTTGACAATTAATCATCGGCTCGTATAATGTGTGGAATTGTGAGCGGATAACAATTTCACACAGGAAACAGTATTCATGTCCCCTATACTAGGTTATTGGAAAATTAAGGGCCTTGTGCAACCCACTCGACTTCTTTTGGAATATCTTGAAGAAAAATATGAAGAGCATTTGTATGAGCGCGATGAAGGTGATAAATGGCGAAACAAAAAGTTTGAATTGGGTTTGGAGTTTCCCAATCTTCCTTATTATATTGATGGTGATGTTAAATTAACACAGTCTATGGCCATCATACGTTATATAGCTGACAAGCACAACATGTTGGGTGGTTGTCCAAAAGAGCGTGCAGAGATTTCAATGCTTGAAGGAGCGGTTTTGGATATTAGATACGGTGTTTCGAGAATTGCATATAGTAAAGACTTTGAAACTCTCAAAGTTGATTTTCTTAGCAAGCTACCTGAAATGCTGAAAATGTTCGAAGATCGTTTATGTCATAAAACATATTTAAATGGTGATCATGTAACCCATCCTGACTTCATGTTGTATGACGCTCTTGATGTTGTTTTATACATGGACCCAATGTGCCTGGATGCGTTCCCAAAATTAGTTTGTTTTAAAAAACGTATTGAAGCTATCCCACAAATTGATAAGTACTTGAAATCCAGCAAGTATATAGCATGGCCTTTGCAGGGCTGGCAAGCCACGTTTGGTGGTGGCGACCATCCTCCAAAATCGGATCTGGAAGTTCTGTTCCAGGGGCCCCTGGGATCCATGGCTGAATCTACATCAACTAAAGCTCATACTGAATCCACTATGAGAACACAGTCTACAGCTTCATTGTTCGCAACAATCACTGGCGCCAGCAAAACGGAATGGTCTTTCTCAGATATCGAATTGACTTACCGTCCAAACACGCTTCTCAGCCTTGGCGTTATGGAGTTTACATTGCCAAGCGGATTTACTGCAAACACGAAAGACACATTGAACGGAAATGCCTTGCGTACAACACAGATCCTCAATAACGGGAAAACAGTAAGAGTTCCTTTGGCACTTGATTTGTTAGGAGCTGGCGAATTCAAATTAAAACTGAATAACAAAACACTTCCTGCCGCTGGTACATATACTTTCCGTGCGGAGAATAAATCATTAAGCATCGGAAATAAATTTTACGCAGAAGCCAGCATTGACGTGGCTAAGCGCAGCACTCCTCCGACTCAGGGCGGCTCCGGTGGTAGCAAACTGGGCGATATTGAATTTATTAAAGTGAACAAATAACTCGAGCGGCCGCATCGTGACTGACTGACGATCTGCCTCGCGCGTTTCGGTGATGACGGTGAAAACCTCTGACACATGCAGCTCCCGGAGACGGTCA

**Expression Product Sequence**

MSPILGYWKIKGLVQPTRLLLEYLEEKYEEHLYERDEGDKWRNKKFELGLEFPNLPYYIDGDVKLTQSMAIIRYIADKHNMLGGCPKERAEISMLEGAVLDIRYGVSRIAYSKDFETLKVDFLSKLPEMLKMFEDRLCHKTYLNGDHVTHPDFMLYDALDVVLYMDPMCLDAFPKLVCFKKRIEAIPQIDKYLKSSKYIAWPLQGWQATFGGGDHPPKSDLEVLFQGPLGSMAESTSTKAHTESTMRTQSTASLFATITGASKTEWSFSDIELTYRPNTLLSLGVMEFTLPSGFTANTKDTLNGNALRTTQILNNGKTVRVPLALDLLGAGEFKLKLNNKTLPAAGTYTFRAENKSLSIGNKFYAEASIDVAKRSTPPTQGGSGGS KLGDIEFIKVNK*

| **Name** | GFP_SpyCatcher | **Source** | (Williams et al., 2018) |
| --- | --- | --- | --- |
| **Resistance** | Ampicillin | **Total plasmid size** | 5760 bp |
| **Parent Vector** | pProEx-Hta | **Seq Primers** | P1 + pBAD rev |
| **Benchling link** | https://benchling.com/s/seq-ayZDRtzOXjYtXTwEvNWS?m=slm-2hjvCkwkuU50ScyzwZU6 | | |

**Description**

GFP SpyCatcher fusion protein for binding to BslA_SpyTag monolayers. Cleavable GST tag.

**Shading Key**

Trc promoter

LacO

His-TEV-GFP-SpyCatcher

**DNA**

TCTGGCAAATATTCTGAAATGAGCTGTTGACAATTAATCATCCGGTCCGTATAATCTGTGGAATTGTGAGCGGATAACAATTTCACACAGGAAACAGACCATGTCGTACTACCATCACCATCACCATCACGATTACGATATCCCAACGACCGAAAACCTGTATTTTCAGGGCGCCATGGGATCCAGTAAGGGTGAAGAACTGTTCACAGGAGTCGTCCCTATCTTGGTTGAGTTAGACGGCGATGTAAATGGTCACAAATTCTCTGTATCAGGGGAAGGCGAAGGCGACGCCACGTATGGCAAACTGACCTTAAAGTTTATTTGCACTACGGGTAAACTGCCGGTGCCATGGCCCACGCTGGTGACCACTCTGACCTATGGTGTTCAGTGTTTCTCCCGTTATCCGGATCACATGAAACAGCACGATTTCTTCAAATCTGCCATGCCGGAAGGCTACGTACAGGAACGGACAATCTTTTTCAAGGACGATGGAAACTACAAAACACGTGCCGAAGTCAAATTTGAAGGAGATACGTTGGTGAATCGCATTGAGCTGAAAGGGATTGATTTTAAAGAAGACGGTAATATCTTAGGTCATAAACTGGAATATAACTACAATAGCCACAATGTGTATATTATGGCGGACAAACAGAAAAACGGAATCAAAGTAAATTTTAAGATTCGCCACAACATCGAAGATGGCAGCGTCCAGTTGGCAGACCACTATCAGCAAAACACCCCTATTGGCGATGGGCCGGTACTCTCACCAGATAACCACTATCTCTCGACACAATCTAAACTGAGCAAAGACCCAAATGAGAAACGCGATCACATGGTTCTGCTGGAGTTCGTTACTGCTGCCGGTATTACCCACGGTATGGACGAACTGTACAAGGGCGGCTCCGGTGGTAGCGCCATGGTTGATACCTTATCAGGTTTATCAAGTGAGCAAGGTCAGTCCGGTGATATGACAATTGAAGAAGATAGTGCTACCCATATTAAATTCTCAAAACGTGATGAGGACGGCAAAGAGTTAGCTGGTGCAACTATGGAGTTGCGTGATTCATCTGGTAAAACTATTAGTACATGGATTTCAGATGGACAAGTGAAAGATTTCTACCTGTATCCAGGAAAATATACATTTGTCGAAACCGCAGCACCAGACGGTTATGAGGTAGCAACTGCTATTACCTTTACAGTTAATGAGCAAGGTCAGGTTACTGTAAATGGCAAAGCAACTAAAGGTGACGCTCATATTAGATCTTAGTGAATTAACAAGCTTGGCTGTTTTGGCGGATGAGAGAAGATTTTCAGCCTGATACAGATTAAATCAGAACGCAGAAGCGGTCTGATAAAACAGAATTTGC

**Expression Product Sequence**

MSYYHHHHHHDYDIPTTENLYFQGAMGSSKGEELFTGVVPILVELDGDVNGHKFSVSGEGEGDATYGKLTLKFICTTGKLPVPWPTLVTTLTYGVQCFSRYPDHMKQHDFFKSAMPEGYVQERTIFFKDDGNYKTRAEVKFEGDTLVNRIELKGIDFKEDGNILGHKLEYNYNSHNVYIMADKQKNGIKVNFKIRHNIEDGSVQLADHYQQNTPIGDGPVLSPDNHYLSTQSKLSKDPNEKRDHMVLLEFVTAAGITHGMDELYKGGSGGSAMVDTLSGLSSEQGQSGDMTIEEDSATHIKFSKRDEDGKELAGATMELRDSSGKTISTWISDGQVKDFYLYPGKYTFVETAAPDGYEVATAITFTVNEQGQVTVNGKATKGDAHIRS*

| **Name** | mCherry_SnC | **Source** | (Williams et al., 2018) |
| --- | --- | --- | --- |
| **Resistance** | Ampicillin | **Total plasmid size** | 5981 bp |
| **Parent Vector** | pProEx-Hta | **Seq Primers** | P1 + pBAD rev |
| **Benchling link** | https://benchling.com/s/seq-M9c864twplzyyNUFbK4o?m=slm-aQfLVfhT2dbx9pTTzRLX | | |

**Description**

mCherry-SnoopCatcher fusion protein for testing of binding to BslA-SnoopTag surfaces. Used for cell-free protein synthesis experiments.

**Shading Key**

Trc promoter

LacO

His-TEV-mCherry-SnoopCatcher

**DNA**

TCTGGCAAATATTCTGAAATGAGCTGTTGACAATTAATCATCCGGTCCGTATAATCTGTGGAATTGTGAGCGGATAACAATTTCACACAGGAAACAGACCATGCATCACCATCACCATCACGATTACGATATCCCAACGACCGAAAACCTGTATTTTCAGGGCGCCATGGGATCCGGAATTCAAAGGCCTACGTCGACGAGCTCAACTAGTGCGGCCGCTTTCGAATCTAGAATGGCTAGCGTGAGCAAGGGCGAGGAGGATAACATGGCCATCATCAAGGAGTTCATGCGCTTCAAGGTGCACATGGAGGGCTCCGTGAACGGCCACGAGTTCGAGATCGAGGGCGAGGGCGAGGGCCGCCCCTACGAGGGCACCCAGACCGCCAAGCTGAAGGTGACCAAGGGTGGCCCCCTGCCCTTCGCCTGGGACATCCTGTCCCCTCAGTTCATGTACGGCTCCAAGGCCTACGTGAAGCACCCCGCCGACATCCCCGACTACTTGAAGCTGTCCTTCCCCGAGGGCTTCAAGTGGGAGCGCGTGATGAACTTCGAGGACGGCGGCGTGGTGACCGTGACCCAGGACTCCTCCCTGCAGGACGGCGAGTTCATCTACAAGGTGAAGCTGCGCGGCACCAACTTCCCCTCCGACGGCCCCGTAATGCAGAAGAAGACCATGGGCTGGGAGGCCTCCTCCGAGCGGATGTACCCCGAGGACGGCGCCCTGAAGGGCGAGATCAAGCAGAGGCTGAAGCTGAAGGACGGCGGCCACTACGACGCTGAGGTCAAGACCACCTACAAGGCCAAGAAGCCCGTGCAGCTGCCCGGCGCCTACAACGTCAACATCAAGTTGGACATCACCTCCCACAACGAGGACTACACCATCGTGGAACAGTACGAACGCGCCGAGGGCCGCCACTCCACCGGCGGCATGGACGAGCTGTACAAAGGCGGCTCCGGTGGTAGCATGAAGCCGCTGCGTGGTGCCGTGTTTAGCCTGCAGAAACAGCATCCCGACTATCCCGATATCTATGGCGCGATTGATCAGAATGGGACCTATCAAAATGTGCGTACCGGCGAAGATGGTAAACTGACCTTTAAGAATCTGAGCGATGGCAAATATCGCCTGTTTGAAAATAGCGAACCCGCTGGCTATAAACCGGTGCAGAATAAGCCGATTGTGGCGTTTCAGATTGTGAATGGCGAAGTGCGTGATGTGACCAGCATTGTGCCGCAGGATATTCCGGCTACATATGAATTTACCAACGGTAAACATTATATCACCAATGAACCGATACCGCCGAAATAATCGTACTACCATCACCATCACCATCACGATTACGATATCCCAACGACCGAAAACCTGTATTTTCAGGGCGCCATGGATCCGGAATTCAAAGGCCTACGTC

**Expression Product Sequence**

MHHHHHHDYDIPTTENLYFQGAMGSGIQRPTSTSSTSAAAFESRMASVSKGEEDNMAIIKEFMRFKVHMEGSVNGHEFEIEGEGEGRPYEGTQTAKLKVTKGGPLPFAWDILSPQFMYGSKAYVKHPADIPDYLKLSFPEGFKWERVMNFEDGGVVTVTQDSSLQDGEFIYKVKLRGTNFPSDGPVMQKKTMGWEASSERMYPEDGALKGEIKQRLKLKDGGHYDAEVKTTYKAKKPVQLPGAYNVNIKLDITSHNEDYTIVEQYERAEGRHSTGGMDELYKGGSGGSMKPLRGAVFSLQKQHPDYPDIYGAIDQNGTYQNVRTGEDGKLTFKNLSDGKYRLFENSEPAGYKPVQNKPIVAFQIVNGEVRDVTSIVPQDIPATYEFTNGKHYITNEPIPPK*
